# Supplementary material for: MetaRibo-Seq measures translation in microbiomes
Source: Nat Commun. 2020 Jun 29;11:3268. doi: 10.1038/s41467-020-17081-z (PMC7324362; doi:10.1038/s41467-020-17081-z)
Supplement: Supplementary file 10 — Supplementary Data 7 [file 41467_2020_17081_MOESM10_ESM.zip › File2/Confidence_High_Taxonomy/409316_out.krona.html]

Javascript must be enabled to view this page.

members
magnitude
magnitudeUnassigned
count
unassigned
taxon
rank

409316\_out

16

superkingdom
16
2

976
16
phylum

16
200643
class

16
171549
order

16
815
family

genus
816
16

species

SRS011084\_contig\_number\_20731SRS014459\_contig\_number\_30487SRS018541\_contig\_number\_12167SRS018888\_contig\_number\_10038SRS043701\_contig\_number\_1245SRS048981\_contig\_number\_3687SRS075021\_contig\_number\_125SRS075773\_contig\_number\_36337SRS077194\_contig\_number\_178SRS104036\_contig\_number\_6997SRS104636\_contig\_number\_428SRS1055099\_contig\_number\_9637SRS143417\_contig\_number\_17338SRS147022\_contig\_number\_674SRS147139\_contig\_number\_36463SRS893279\_contig\_number\_818
16
817
